# A Few Other Charities

**Published:** 1893-12-23

**Authors:** 


### A FEW OTHER CHARITIES.

**British Home for Incurables.**—Helpless, hopeless, homeless. The Board of Management of the British Home for Incurables earnestly appeal for 500 new annual subscribers of one guinea each, to counterbalance the loss of income sustained by selling out capital to build the new home, now nearing completion, at Streatham. In addition to maintaining the suffering inmates of the present home at Clapham, the charity has nearly 300 pensioners, each receiving £20 a year. Applications from all parts of the United Kingdom are constantly being received. Cheques and postal orders to be crossed "Barclay and Co.," and sent to Mr. R. G. Salmond, Secretary, 73, Cheapside, E.C.

**City of London Truss Society,** 35, Finsbury Square.—For the relief of the ruptured poor throughout the kingdom. At the present time nearly 10,000 of both sexes and all ages are treated annually, while over 490,000 patients have been relieved since its formation eighty-five years ago. Secretary, Mr. John Whittington.

**The Hampstead Home Hospital and Nursing Institute** has made steady progress during the twelve years of its existence. Commencing with one house, it now comprises a block of four houses, with a separate house, connected by a covered way, for the private nursiug staff. During the past year nearly 200 patients have been treated, in addition to a large number of emergency cases. This institution deserves more local support, as the majority of the patients only pay a nominal fee, all accidents being admitted free. Hon. Secretary, Mr. R. A. Owthwaite.

**Homes for Little Boys,** Farningham and Swanley, Kent.—These homes are caring for 500 homeless and orphan lads, who are received from all parts of the United Kingdom, taught trades, and fitted for a useful life. The committee have just issued an appeal, in which they state that if the income is not considerably increased they must stop taking in any more boys, and must curtail their work. They feel sure, however, that a generous public will not let this happen. Contributions of any amount will be most thankfully received at the offices, Bank Buildings, Ludgate Circus, E.C. Secretaries, Messrs. Arthur E. Charles and William Robson.

**Irish Distressed Ladies' Fund,** 17, North Audley Street, W. Patron, the Queen.—This association helps those ladies of Ireland who depend for their support on the proceeds of Irish property, but who, owing to the rent difficulty and causes beyond their own control, have been reduced to absolute poverty. Relief is given independently of any question of politics or religion; employment is found, when possible, for those able to work; pecuniary help is given to the aged and infirm; and the education of children is paid for. Secretary, Lieut.-General W. M. Lees.

**London Orphan Asylum,** Watford. Instituted 1813.—The commercial depression, still so prevalent, has naturally made itself felt in the form of diminished receipts, compelling a loan of £2,000 from the bankers, and a reduction in the number of the children to be elected, a step the more to be regretted as the candidates seeking relief are as numerous as ever. Under these circumstances the managers earnestly invite the kindly interest and support of the public towards the maintenance of the 505 orphans now under the care of the institution, and in order that larger admissions may be reverted to at the earliest possible period. 88 orphans have been admitted this year; 5,621 orphans have been already benefited. Donations will be very gratefully received by the Secretary, Mr. James Rogers, at the office, 21, Great St. Helens, E.C.

**Metropolitan Drinking Fountain and Cattle Trough Association.**—This is the only society which provides free supplies of water for the many thousands of men, women and children, besides horses, cattle, sheep, and dogs that are daily toiling through our streets. It depends entirely on voluntary contributions, which are urgently needed. Secretary, Mr. M. W. Milton, 70, Victoria Street, Westminster, S.W.

**National Health Society.**—The basis of the whole system of instruction which this most useful society comprises, may be said to lie in the proverb that "Prevention is better than cure." It goes straight to the root of the matter, as its aim is to diffuse by all possible means sanitary knowledge of every kind, comprising home nursing, rearing of infants, prevention of the spread of infectious diseases, and by no means least, important lectures on sanitary reform in food and cookery. The society gives courses of lectures on the various subjects which affect health, not only in London and its vicinity, but even so far afield as Glasgow and Aberdeen. We wish this useful society all success in its work. Secretary, Miss Lankester, 53, Berners Street, W.C.

**Orphan Working School,** Haverstock Hill and Hornsey. Offices, 73, Cheapside, E.C. Founded 1758.—This is a national and unsectarian institution which maintains over 500 children, varying in age from infancy to fourteen or (in special cases) fifteen years. It is greatly in want of funds at the present time, as £8,000 is needed to pay off the debt to the bankers. This orphanage is the oldest one of the kind in the metropolis, and over 90 per cent. of the old scholars turn out satisfactorily. Secretary, Algernon C. P. Coote, M.A.

**Metropolitan and National Nursing Association,** 23, Bloomsbury Square, W.C.—Hon. Secretary, Rev. Dacre Craven; Lady Superintendent, Miss Hughes.

**Zenana, Bible, and Medical Mission,** 2, Adelphi Terrace, W.C.—This society has a threefold object in its work, addressing itself to the spiritual, mental, and bodily evils under which the unhappy women of India have groaned for centuries. It is worthy of help from all. Especially should it appeal to women, for their sisters in India can only be reached by women; their sufferings only alleviated by women; and only women can enter their homes and personally influence them in mind and body. The great need of the mission at the present time is for friends of the work to subscribe £25,000 to enable the Council to consolidate the Zenana school and medical work of this useful society. So energetic is the society that we hope the generosity of English men and women will enable them to do much more than it is possible for it to attempt with the present inadequate income. Hon. Finance Secretary, Mr. W. T. Paton; General Secretary, Rev. A. R. Cavalier.

The following are deserving institutions, to the work of which we would also call the attention of the charitable:—

London Temperance Hospital, Hampstead Road, N.W.—In-patients, 1892, 859; out-patients, 1892, 4,107 new cases; casualties 1892, 4,083, new cases. Funds urgently needed to meet large increase of work. Secretary, Mr. E. W. Taylor; Matron, Miss S. E. Orme.

### Consumption.

Royal National for Consumption, Ventnor.—Secretary, Mr. E. Morgan, 34, Craven Street, W.C.; General Superintendent, Major Payne; Matron, Miss Busby.

### Children.

Alexandra Hospital for Children with Hip Disease, Queen Square, Bloomsbury, W.C.—Sixty-eight cots, always full. Funds urgently needed. Mr. Stanley Smith, Secretary; Miss C. E. Moore, Matron.

Cheyne Hospital for Sick and Incurable Children, Cheyne Walk, Chelsea.—President, H.R.H. the Princess of Wales. Fifty beds, always quite full. Children may remain for an indefinite period. Two-thirds of cases received during last fifteen years discharged cured or greatly relieved. Funds much needed to meet winter's expenditure. Visitors welcomed week-day afternoons. Secretary, Mr. Reginald Blunt; Lady Superintendent, Miss Elam.

Hospital and Home for Incurable Children, 2, Maida Vale, W.—Matron, Miss Coleman.

### Fever.

London Fever Hospital, Liverpool Road, N.—Secretary, Major W. Christie; Matron, Miss H. F. Ingall.

